# Detection of Enteric Viruses and Core Microbiome Analysis in Artisanal Colonial Salami-Type Dry-Fermented Sausages from Santa Catarina, Brazil

**DOI:** 10.3390/foods10081957

**Published:** 2021-08-22

**Authors:** Roberto Degenhardt, Doris Sobral Marques Souza, Leidiane A. Acordi Menezes, Gilberto Vinícius de Melo Pereira, David Rodríguez-Lázaro, Gislaine Fongaro, Juliano De Dea Lindner

**Affiliations:** 1 Food Technology and Bioprocess Research Group, Department of Food Science and Technology, Federal University of Santa Catarina (UFSC), Florianópolis 88034-000, SC, Brazil; roberto.degenhardt@gmail.com (R.D.); doris.sobral@gmail.com (D.S.M.S.); leidianeacordi@gmail.com (L.A.A.M.); 2 Biological and Health Sciences Department, West of Santa Catarina State University (UNOESC), Joaçaba 89600-000, SC, Brazil; 3 Laboratory of Applied Virology, Department of Microbiology, Immunology and Parasitology, Federal University of Santa Catarina (UFSC), Florianópolis 88034-000, SC, Brazil; gislainefongaro@gmail.com; 4 Neoprospecta Microbiome Technologies, Sapiens Park, Florianópolis 88056-000, SC, Brazil; 5 Department of Bioprocess Engineering and Biotechnology, Federal University of Paraná (UFPR), Curitiba 80060-000, PR, Brazil; gilbertovinicius@gmail.com; 6 Microbiology Division, Faculty of Sciences, University of Burgos, 9070 Burgos, Spain; drlazaro@ubu.es; 7 Center for Emerging Pathogens and Global Health, University of Burgos, 9070 Burgos, Spain

**Keywords:** swine and pork production chain, Hepatitis E virus, Rotavirus-A, metagenomic analysis, food safety

## Abstract

Microbial fermentation plays an important role in the manufacturing of artisanal sausages and can have major effects on product quality and safety. We used metagenomics and culture-dependent methods to study the presence of Hepatitis E virus (HEV) and Rotavirus-A (RV-A), and fungal and bacterial communities, in artisanal Colonial salami-type dry-fermented sausages in Santa Catarina state, Brazil. Lactic acid bacteria (LAB) and yeast dominated the microbiome. *Latilactobacillus sakei* and *Debaryomyces hansenii* were ubiquitous and the most abundant species. The DNA of some foodborne pathogens was found in very low concentrations although viable cells of most of these species were undetectable by cultivation methods. The characteristics of the raw material and hygiene of the artisanal sausage manufacturing process resulted in high loads of beneficial microorganisms and the absence of HEV and RV-A viruses as determined by RT-qPCR assays. In conclusion, high LAB load in sausages was more relevant to preventing pathogen growth than the ripening time and/or physicochemical characteristics. However, the presence of *Clostridium* spp. and other pathogens in some samples must be taken into account for the development of future preservation methods; appropriate LAB starter cultures and health surveillance are required in the production process to prevent foodborne outbreaks.

## 1. Introduction

Fermented sausages (dry and semi-dry) make up a substantial proportion of pork produced and consumed in western Santa Catarina State. This is in part a consequence of the European origin and cultural characteristics of the population in this region in the south of Brazil. Several small and medium-scale production companies are dedicated to the transformation of pork meat, employing artisanal production techniques including natural fermentation. However, these production practices can allow the survival and multiplication of spoilage and pathogenic microorganisms if hygiene and sanitary conditions and process parameters are not strictly controlled.

There is a great diversity of dry-fermented sausages produced in the world. Different raw meat types, formulations, additives, processes, casing, and drying periods are used [1]. Colonial sausages produced in Santa Catarina State are characterized by low acidity and high moisture content when they are offered for sale, but they continue the ripening process outdoors in the market, gradually losing moisture [2]. These uncontrolled conditions can lead to a risk for the multiplication of undesirable microorganisms.

The production of artisanal sausages involve fermentation by the indigenous microbiota of meat (composed mainly by lactic acid bacteria—LAB) producing organic acids, and consequently a decrease in the pH that allows preservation of the product. *Lactobacillus*, *Pediococcus*, *Enterococcus*, and *Leuconostoc* are believed to be the principal genera involved in sausage fermentation [3,4], and diverse LAB are found in food products according to the manufacturing practices used. The preservation ability of LAB is based on competition for nutrients and the production of antimicrobial metabolites, hydrogen peroxide and bacteriocins. The selective influence of diverse factors (e.g., pH, redox potential, water activity (a_w_), temperature, relative humidity, oxygen availability) in fermented sausage will contribute to determining the microbial consortium present.

The microbial communities in fermented sausage play significant roles in the flavor, texture, quality, and safety of sausages [5,6]. Understanding the ecology of fermented sausages is clearly key to understanding the physical and chemical changes that occur during fermentation and ripening [7]. High-throughput sequencing of the microbiota from salami-type dry-fermented sausages of different origins indicates that LAB and coagulase-negative cocci, including both micrococci and coagulase-negative staphylococci (CNS) are the predominant groups in spontaneously fermented artisanal sausages [8]. *Debaryomyces, Penicillium*, and *Aspergillus* are the yeast and mold genera most commonly found [6,9,10,11]. Studies involving metagenomics for this type of fermented product are scarce in Brazil, and therefore, the ecology of Brazilian artisanal dry-fermented sausages needs further investigation.

Hepatitis E virus (HEV) and Rotavirus—A (RV-A) belong to the human enteric viruses group. They are non-enveloped and icosahedral viruses, and are very persistent in the environment [12]. HEV has a positive-sense single-stranded RNA and RV-A a double-stranded and segmented RNA (11 segments) that facilitates reassortment events among species [12]. Both are zoonotic viruses that infect many animal species as well as humans. They are mainly transmitted by the fecal-oral route, although HEV is also transmitted parenterally (rare), or by the ingestion of contaminated meat from viremic animals [12,13].

HEV and RV-A infect domestic pigs, which serve as reservoirs, and are emerging risks for human infection [13,14]. These viruses are excreted at high concentrations in animal stools (mainly from swine) so animal waste can contaminate water supplies, and when used in crops may contaminate food. Indeed, they persist as infectious particles in the environment, e.g., water and sewage, for several weeks at lower than body temperatures [13,15,16,17]. HEV circulation has already been reported in humans and domestic swine in Brazil [17,18,19].

The present study aimed to detect and quantify HEV and RV-A in artisanal Colonial salami-type dry-fermented sausages. We also analyzed the presence of fermenting, spoiling, and pathogenic microorganisms in the sausages. Both culture-dependent and -independent (metagenomic) approaches were used.

## 2. Materials and Methods

### 2.1. Colonial Salami-Type Dry-Fermented Sausage Production

Colonial salami-type dry-fermented sausages were collected from 13 production sites (Table 1) under the state inspection system (SIE) in the Vale do Rio do Peixe zone, Santa Catarina state, Brazil (ca. 27°10’ S; 51°30’ W). The sausages were prepared using the same manufacturing process in all sites. Pigs were slaughtered and the carcasses underwent temperature equalization for up to 24 h in cooling chambers to establish rigor mortis. The carcasses were exposed and shanks and palettes were used to prepare the sausages. The meat was comminuted in a meat grinder and seasoned with NaCl, curing agent (NO_2_), and spices.

The production of these sausages does not involve addition of commercial starter cultures for pan-curing. The fermentation is due to the natural microbiota of the meat and environment. After homogenization in a mixer, the meat is extruded into a natural bovine or a synthetic cellulose casing. The sausages are placed in a smoker where the drying is started, and the curing is completed under smoking. After smoking, the sausages are ready for sale. Thereafter, drying took place in the market, subject to the environmental conditions of the market stall site. The 13 sausage samples used had drying times of between one and 40 days, with half of the samples having been dried for more than seven days.

### 2.2. Physical-Chemical Evaluation

The physical-chemical characteristics of the sausages (Table 1) were determined using the official methods of the Ministry of Agriculture, Livestock and Supply (MAPA) [20]. The pH was determined using a potentiometer (Quimis, model Q400A, Diadema, SP, Brazil). The moisture content was determined by a gravimetric method, placing the samples in a drying oven (SP Labor, model SP-100/150, SP, Brazil) at 105 °C for 24 h [21]. The NaCl content was determined by the mercury metric method [20]. The a_w_ was determined by grinding the samples and placing them into a digital hygrometer at 25 °C (Aqualab Model-Series 3 TE, Decagon Devices Inc., Pullman, USA). Water phase salt (WPS) was calculated from the values of NaCl and moisture content, according to Koral and Köse [22], using the following equation:

WPS% = (NaCl%/(NaCl% + moisture%)) × 100

### 2.3. Microbiological Analyses

The samples were tested for several microbial groups: total coliforms (TC), thermotolerant coliforms (TTC), coagulase-positive staphylococci (CPS), sulfite-reducing clostridia (SRC), LAB, Enterococci, *Salmonella* and *Listeria monocytogenes.* TC were counted by spread plating on Violet Red Bile Lactose agar (VRBL, Oxoid Limited, Hampshire, United Kingdom), incubated aerobically at 37 °C for 24 h [23]; TTC by pour plating on double-layered VRBL agar, incubated aerobically at 44 °C for 24 h; CPS by spread plating on Baird-Parker agar (BPA, Neogen, Lansing, MI, USA), incubated aerobically at 35 °C for 24–48 h [24]; SRC by pour plating on Iron Sulfite agar (ISA, Neogen), incubated anaerobically at 37 °C for 24–48 h [25]; LAB by pour plating on De Man, Rogosa and Sharpe agar (MRS, Neogen), incubated anaerobically at 30 °C for 72 h [26]; Enterococci by spread plating on m-Enterococcus agar (Neogen), incubated anaerobically at 35 ± 2 °C for 24–48 h; *Salmonella* by incubating a 25 g pre-enrichment sample in buffered peptone water (BPW, Neogen) at 34–38 °C for 18 h, selective enrichment by adding 0.1 mL of the BPW to modified semi-solid Rappaport-Vassiliadis medium (MSRV, Neogen) at 41.5 ± 1 °C for 24 h and 1 mL of BPW to Muller-Kauffmann Tetrathionate-Novobiocin broth (MKTTn, Neogen) at 37 ± 1 °C for 24 h, plating-out in xylose lysine deoxycholate agar (XLD, Neogen) at 37 °C for 24 h and brilliant green agar (BGA, Neogen) at 37 °C for 24 h, with confirmation by biochemical and serological testing [27]; *Listeria* spp. by incubating a 25 g pre-enrichment sample in demi Fraser broth (DF, Neogen) at 30 °C for 24 h, selective enrichment by incubating 0.1 mL of the DF in Fraser broth (Neogen) at 35 ± 1 °C for 24–48 h, then plating-out on Listeria agar (ALOA, Laborclin, Pinhais, Paraná, Brazil) at 37 °C for 24 h, with confirmation by biochemical testing [28].

Yeasts and molds were counted by spread plating on dichloran glycerol agar (DG18, Neogen) containing chloramphenicol (0.1 g/L) and incubating at 25 °C for 5–7 days [29]. Macroscopic observation was used to distinguish between yeasts and molds among the colonies growing on the plates.

### 2.4. Viral Detection by RT-qPCR

Virus was detected by mechanical disruption of tissues followed by silica-membrane RNA extraction, as described by García et al. [14] and Rodríguez-Lázaro et al. [30]. Briefly, 25 g samples of sausage (Table 1) were collected, chopped using a sterile scalpel blade and homogenized; aliquots of 0.25 g were taken from the samples, and they were inoculated with ~10^5^ PFU (Plaque Forming Units) of MNV-1 (Murine Norovirus-1) as sample process control virus (SPCV). Each sample (including SPCV) was homogenized in 1 mL of lysis buffer RLT (Qiagen, Hilden, Germany) containing 0.14M β-mercapto-ethanol in a tissue homogenizer, and incubated for 5 min at room temperature. The samples were then centrifuged at 10,000× *g* for 20 min at 4 °C and the supernatants (800 µL) were transferred to new tubes for nucleic acid extraction with the RNeasy^®^ Mini kit (Qiagen, Germany), following the manufacturer’s protocols. The resulting nucleic acid samples were subjected to RT-qPCR, both undiluted and diluted tenfold to reduce the inhibitory effects of any contaminants in the samples.

The TaqMan technique was used for RT-qPCR, as previously described by Baert et al. [31] for MNV-1 as SPCV, Jothikumar et al. [32] for HEV and [33] for RV-A. All amplifications were performed in a StepOne Plus^®^ Real-Time PCR System (Applied Biosystems, Waltham, MA, USA), and each sample was analyzed in triplicate. Ultrapure water was used as the non-template control for each assay.

### 2.5. Metagenomic Analyses

DNA was extracted from samples with the DNeasy Power Soil Pro kit (Qiagen, Germany), following the manufacturer’s instructions. Total DNA extracted was used as the template for Next Generation Sequencing (NGS), on the Illumina MiSeq platform (Illumina Inc., San Diego, CA, USA).

The microbiome of the samples was studied by high throughput sequencing for bacterial taxonomic identification based on conserved and variable regions V3/V4 of the 16S rRNA gene with primers 341F (CCTACGGGRSGCAGCAG) and 806R (GGACTACHVGGGTWTCTAAT) [34,35]. Extracted DNA was subjected to PCR using a previously described protocol [36] developed by Neo-prospecta Microbiome Technologies (Florianopolis, Brazil). The first PCR used sequences based on Illumina’s TruSeq adapters, which allows a second PCR with primers with indexed sequences. PCRs were carried out in triplicate using Taq Platinum (Invitrogen, Waltham, MA, EUA) with the following conditions: PCR1—95 °C/5 min, 25 cycles (95 °C/45 s, 55 °C/30 s, 72 °C/45 s) and final extension 72 °C/2 min; PCR2—95 °C/5 min, 10 cycles (95 °C/45 s, 66 °C/30 s, 72 °C/45 s) and final extension 72 °C/2 min.

The community of filamentous fungi and yeasts was studied by amplifying the ITS1 region using ITS1F (GAACCWGCGGARGGATCA) and ITS2R (GCTGCGTTCTTCATCGATGC) primers [37], and the same conditions for PCR1. For PCR2, the conditions were: 95 °C/5 min, 15 cycles (95 °C/45 s, 66 °C/30 s, 72 °C/45 s) and final extension 72 °C/2 min. The final reaction products were purified using a protocol developed by Neoprospecta involving magnetic beads. The samples were grouped in libraries quantified by Real-Time PCR using KAPA Library Quantification (KAPA Biosystems, Woburn, MA, USA). Library pools were sequenced in a MiSeq sequencer system (Illumina Inc., USA) using V2- 300 cycle kit, in a paired-end run, without normalization of libraries. The raw data (DNA sequences in fastq files) were analyzed through bioinformatics workflow considering accumulated error in the sequencing to be at most 1%. To identify the species of microorganisms present in the samples, the DNA sequences obtained were compared with a proprietary database containing well-characterized DNA sequences.

Sequencing data for each sample were processed with the Quantitative Insights into Microbial Ecology (*Qiime*) software package [38]. The sequencing output was analyzed by a read quality filter; reads with an average Phred score < 20 were removed and then 100% identical reads were clustered. Clusters with fewer than five reads were excluded from further analysis, to remove putative chimeric sequences. The remaining good-quality sequences were further clustered at 97% similarity to define operational taxonomic units (OTU). OTUs were classified by comparison with a custom 16S rRNA/ITS1 database (NEORefDB, Neoprospecta). Sequences with at least 99% identity to the reference database were taxonomically assigned. Each OTU was given an arbitrary unit corresponding to the relative abundance (r.a.) calculated as the number of reads pertaining to each species as a percentage of the total number of sequences read for each sample. Species with a r.a. below 1% were grouped in the category “others”. The r.a. values were compared between OTUs using heatmaps prepared on *Qiime*.

### 2.6. Statistical Analyses

Differences in virus parameters between samples collected from different sites and seasons were analyzed by nonparametric analysis of variance (Kruskal–Wallis) performed with Statistic 7.0. Pearson correlation and linear regression tests, ANOVA, and Student’s *t*-tests were performed using GraphPad Prism 5.0 (USA). Differences were considered statistically significant at a *p*-value ≤ 0.05.

## 3. Results and Discussion

### 3.1. Physical-Chemical Characteristics

Selected physical-chemical characteristics (pH, moisture, NaCl, WPS and a_w_) of the sausage samples are summarized in Table 1. The sausages can be classified into two subgroups according to the criteria of Brazilian legislation [39]: (i) dry sausages, with moisture ≤ 40% and a_w_ ≤ 0.92, and (ii) semi-dry sausages, for which these parameters are not stipulated. A particular feature of these locally produced sausages is that they are offered for sale as semi-dry sausages and, due to the environmental conditions, they start to present dry sausage characteristics as the ripening continues. The dry sausages (L01, L04, L07, L10, L12) had undergone seven or more days of ripening, whereas the semi-dry sausages (L02, L03, L05, L06, L08, L09, and L11) had undergone up to six, except for samples L02 and L06; these two sausages had been maintained under refrigeration at the site of sale, reducing dehydration.

All the samples presented pH between 5.1 and 6.4 (Table 1), characteristic of the sausages produced in the region. This weak acidity is also characteristic of Italian sausages, produced in the Mediterranean region only with pork meat. Low pH and a_w_ are the two main obstacles to spoilage and the development of pathogenic microorganisms in fermented sausages [40]. If these factors are weak, as is the case in our samples due to the relatively high pH of the samples (≥5.1), the association between a_w_ and the competitive microbiota (mainly LAB) becomes very important to the safety of the product. The WPS is not frequently used to assess the potential for multiplication of spoilage and/or pathogenic microorganisms in meat sausages in Brazil, but it is a potentially valuable indicator for small producers, due to its relationship with water activity.

The minimum salt concentration in the samples was 3.01 (sample L08) and the maximum was 6.15 (sample L04). The upper limit of sensorial consumer acceptability for salt in cured meats is 3.5% to 5.0% with 1.5% to 2.0% being optimal. The salt concentration was therefore acceptable in the samples, except for sample L04. The higher concentration of salt in this case appears to be a consequence of excessive drying: the moisture content was only 30.2%. In artisanal cured products, salt is still the main resource used for conservation, and this is the main reason for the high salt content in these sausages, mostly between 3.7% and 5.04% (Table 1). These values are compatible with those required for the preservation of sausages and related products [41].

### 3.2. Viral Detection

The samples of artisanal Colonial salami-type dry-fermented sausages from different production sites (Table 1) were tested by RT-qPCR for HEV and RV-A. Neither virus was detected in any of the sausage samples, and the mean viral extraction efficiency (measured as SPCV -MNV-1 recovery) was 30%.

HEV is more frequently detected in feces than serum and liver samples from infected animals [42], but there is nevertheless a risk of foodborne hepatitis E infection from consumption of pork products. Boxman et al. [43] found HEV RNA in 14.6% of pork sausages collected from a Dutch market from 2017 to 2019, and HEV RNA was detected in 18.5% of salami (*n* = 92) samples; sequence-based typing was successful for 33 samples, and all were genotype 3c. Berto et al. [44] found HEV RNA in four samples of liver sausage in France, and three of them had infectious particles of HEV as assessed by cell culture assay. Colson et al. [45] found HEV RNA of genotype 3 in seven samples of raw figatelli purchased in French supermarkets. However, HEV RNA was not detected in any of the 22 sausages from different production sites analyzed in this work. This contrasts with Souza et al. [17], who detected HEV in effluent after swine manure digestion by psychrophilic anaerobic biodigesters (PABs) in Concórdia city, in Santa Catarina state. This demonstrates the circulation of HEV in the pig-finishing farms in the zone where the Colonial sausage we studied was produced.

Lowering pH during meat fermentation is believed to inhibit the growth of certain pathogens. Wolff et al. [46] tested the pH resistance of a genotype 3 strain of HEV. Only minimal inactivating effects were found at pH conditions common in sausages during curing (4.5 to 6.5), simulated using D/L-lactic acid. High salt concentrations are also used to preserve meat products and to inactivate foodborne pathogens. Wolff et al. [47] tested the effects of NaCl, sodium nitrite, and sodium nitrate concentrations on the infectivity of HEV. Conditions consistent with those in fermented sausages were simulated. Treatments with up to 20% NaCl for 24 h at 23 °C, with and without the addition of 0.015% nitrite or 0.03% nitrate, did not inactivate the virus, demonstrating that HEV is highly stable at salt concentrations used to preserve raw meat products. Therefore, the acid pH and high salinity of the products we tested were unlikely to be responsible for the absence of HEV.

Organs from naturally HEV-infected pigs were tested by García et al. [14] and the HEV virus was not detected in loin samples: meat even from infected animals may therefore be only a low risk for HEV transmission. The main ingredient used in the preparation of Colonial salami is the loin, and this may explain the absence of HEV RNA from the samples tested, despite evidence of the circulation of HEV in pig farms in the region [17]. Other organs would need to be analyzed to assess the HEV contamination of the pigs in the region.

RV-A RNA was not detected in the Colonial sausage samples analyzed, evidencing the good sanitary conditions during slaughter and sausages preparation. RV-A zoonotic transmission to humans usually occurs via direct contact with infected animals or fomites [15]. RV-A causes diarrheic diseases in swine and meat contamination could occur in the slaughterhouse or during sausage preparation by handlers and fomites. RV-A is the most prevalent rotaviral cause of diarrhea in swine [48]. Other RV species that can infect pigs were not investigated in this work.

### 3.3. Microbiological and Metagenomic Analyses

The combined culture-dependent and -independent approaches are best for analyses of the microbiota of fermented foods [49]. Culture-dependent analysis showed high levels of LAB and yeasts and the presence of enterococci and molds in all samples. There were low or undetectable counts of undesirable microbiota, in accordance with Brazilian standards (Table 2). In general, the samples showed similar cell count values that were independent of the ripening time. Likewise, there was no correlation between ripening time and microbiota in the metagenomic analyses. The pH in all samples was higher than 5.0, indicating that LAB community was mostly bacteria with low acid production capacity, resulting in low acidity fermented sausages. Although low acidity does not favor the inhibition of undesirable microbiota, it enhances the water-retention capacity of meat proteins, contributing to the maintenance of moisture during ripening [50]. The yeast community is important for the development of flavor and inhibition of toxigenic molds.

**Figure 1:**
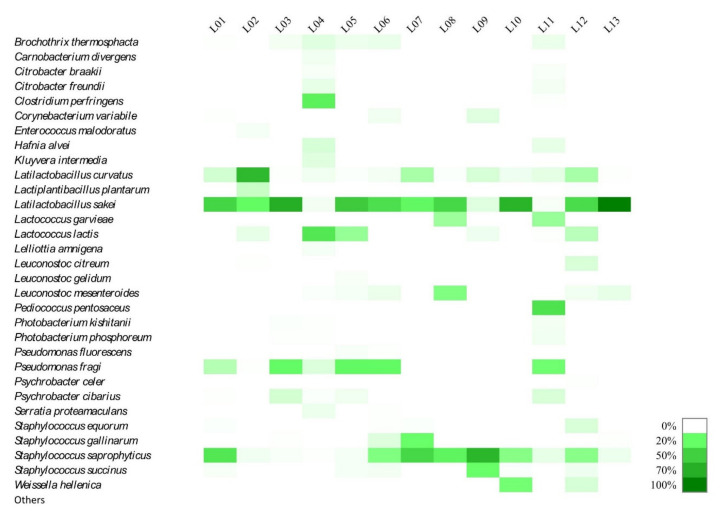
Relative abundance (%) of bacterial species in artisanal Colonial salami-type dry-fermented sausages. Species with a very small number of reads (relative abundance < 1%) were classified into the category “others”.

The 16S amplicon target sequencing identified more than 60 species each with a r.a. above 0.1% (Figure 1 shows the 31 species with over 1% r.a., in one or more samples). Species with a very small number of reads (r.a. < 1%) were classified into the category “others”.

LAB and CNS were the most abundant groups in our samples (Figure 1), consistent with previous reports for dry sausages from Spain and Italy [6,8]. LAB was predominant in 11 of the 13 sausage samples: *Latilactobacillus sakei* was the most abundant taxon in samples L01, L03, L05, L06, L08, L10, L12, and L13 (r.a. of 43.56%, 65.85%, 49.57%, 38.38%, 42.70%, 62.75%, 40.30%, and 92.14%, respectively); *Latilactobacillus curvatus* was predominant in sample L02 (r.a. 60.31%); *Lactococcus lactis* in sample L04 (r.a. 33.33%), and *Pediococcus pentosaceus* in sample L11 (r.a. 36.39%).

Most samples (Figure 1) contained three or four species with r.a. above 5%, although in sample L13 *Lb. sakei* was massively predominant and the microbial community was not very diverse. *Lb. curvatus* was found at r.a. of between 5% and 12% in samples L01, L07, L09, and L12, *Lactococcus lactis* at between 9% and 14% in samples L05 and L12, *Lactococcus garvieae* at around 13% in samples L08 and L011, *Leuconostoc mesenteroides* at 17% in L08, *Lactiplantibacillus plantarum* at 7% in L02 and *Weissella hellenica* at 18% in L10. *Lb. sakei, Lb. curvatus* and *Lp. plantarum* are the species of LAB commonly found in sausages with no added starters. Other species of *Weissella, Leuconostoc*, *Lactococcus*, and *Pediococcus* were also found, but as part of the subdominant microbiota in our samples. The fermentation in these sausages is spontaneous, so the microorganisms detected come from the meat and the surrounding environment [6].

The manufacturing process of artisanal Colonial salami-type dry-fermented sausages from Santa Catarina includes spontaneous fermentation, cure and drying as conservation. Thus, the obstacles to the development of spoilage and pathogenic microbiota are the addition of preservatives (NaCl and sodium nitrite), pH, a_w_, and the competitive microbiota, mostly LAB. The protective effects of LAB are due in part to their production of bacteriocins with antimicrobial activity against pathogens [51]. Hebert et al. [52] showed that *Lb. curvatus* CRL705 isolated from Argentinean artisanal fermented sausage produces bacteriocin with anti-listeria activity. Palavecino et al. [6] describe coagulase-negative staphylococci (CNS) with antimicrobial activity. The presence of CNS (*Staphylococcus saprophyticus*, *Staphylococcus galinarum*, and *Staphylococcus equorum*) in our samples (Figure 1) may be one of the reasons for the absence of pathogens common in meat products, such as *Clostridium botulinum*, *Staphylococcus aureus*, *Listeria monocytogenes*, and *Escherichia coli* [53].

In general, the sausage samples (Table 2) that showed the best hygiene indicator results (TC, TTC, CPS, SRC, and *Salmonella*) were drier (e.g., L10) and had longer ripening time (e.g., L02). This is in contrast to the observation for the high LAB semi-dry sausage subgroup, which corroborates the metagenomic data (Figure 1). *Listeria* spp. were detected in 38.4% of the samples, but *L. monocytogenes* was not detected at a significant r.a. in any sample (Figure 1). *Listeria* spp. are detected in many fermented sausages with low acidity and high a_w_ [54,55,56]. *Listeria* spp. counts determined by culture-dependent methods are generally low in fermented sausages [57,58], and culture-dependent methods employ enrichment and cell concentration steps. Therefore, the possibility of isolating a microorganism is greater than for the metagenomic analysis when it is present at low r.a.

In addition to LAB, species of the *Micrococcaceae* family, mainly belonging to the genus *Staphylococcus*, are commonly found in artisanal sausages. *S. saprophyticus, Staphylococcus xylosus*, *S. equorum*, and *Staphylococcus carnosus* are often predominant. *Staphylococcus succinus* is also frequently observed in sub-dominant populations [7,59]. For samples L07 and L09 (Figure 1), the predominant microorganism was *S. saprophyticus* (r.a. 42.60% and 61.65%, respectively). This species was also found in samples L01, L06, L08, L10, and L12 (r.a. from 15% to 26%). *S. succinus* was found in sample L09 at an r.a. off 20%.

*Brochothrix thermosphacta, Carnobacterium* spp., clostridia, and *Pseudomonas* spp. are among the bacteria most frequently causing spoilage of refrigerated pork meat [60]. *Pseudomonas fragi* (Figure 1) was found in samples L01, L03, L05, L06, and L011 with r.a. between 10% and 23%. *Carnobacterium divergens* (r.a. 1.87%) and *Clostridium perfringens* (r.a. 27.76%) were found in sample L04, in which the abundance of lactobacilli was low. *B. thermosphacta* was detected in samples (Figure 1) with a low abundance of lactobacilli (L03, L05, and L11). However, for sample L09, *S. saprophyticus* was predominant, and although the r.a. of lactobacilli were low, no sequences belonging to *B. thermosphacta*, *C. divergens* (r.a. 1.87%), *Pseudomonas* spp. or clostridia were detected.

**Figure 2:**
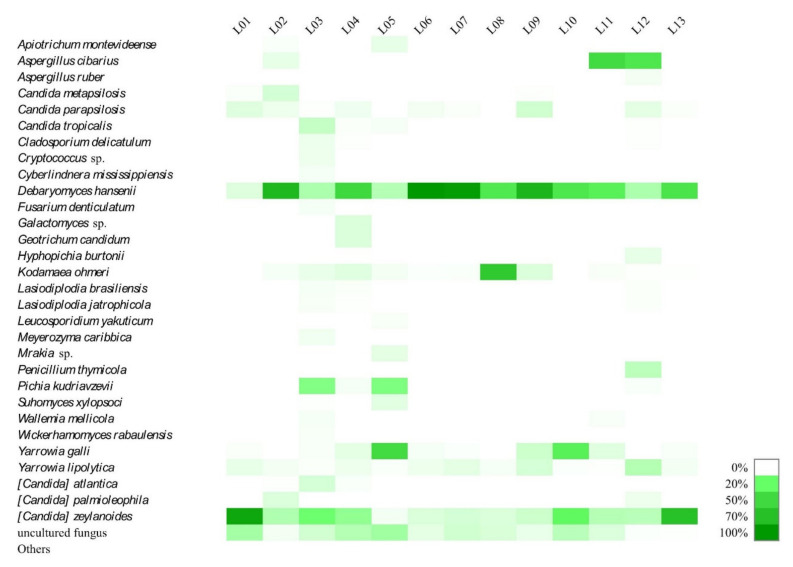
Relative abundance (%) of fungal species in artisanal Colonial salami-type dry-fermented sausages. Species with a very small number of reads (relative abundance < 1%) were classified into in the category “others”.

Taxonomical assignment of the yeasts (Figure 2) showed the dominance of *Debaryomyces hansenii* in L02, L04, L06, L07, L09, L10, L11, and L12 (r.a. 63.49%, 44.25%, 82.44%, 80.01%, 65.71%, 35.20%, 28.92% and 10.85%, respectively) followed by *Candida zeylanoides,* predominant in samples L01, L03, and L12 (r.a. 73.91%, 19.13%, and 58.23%, respectively), *Yarrowia galli* predominant in L05 (r.a. 42.15%), and *Kodamaea ohmeri* in L08 (r.a. 53.31%). *D. hansenii*, *C. zeylanoides* and *Yarrowia lipolytica* were found in all the samples, although they were less abundant than the other yeast groups. *Y. galli, Pichia kudriavzevii, K. ohmeri, Candida parapsilosis* and *Candida tropicalis* were also found with relevant r.a. in the sub-dominant populations of the samples.

*D. hansenii* is the most common yeast in fermented sausages and contributes to two important characteristics: production of volatile compounds during ripening, especially esters, branched alcohols, and aldehydes [61,62], and bioprotective antifungal activity against toxigenic penicillia [63]. Species belonging to the genera *Candida, Yarrowia*, and *Pichia* are also frequently found [6,51,64].

Development of mold on the surface of the casings during ripening of fermented sausages is common, and molds are present in the environment at the production and ripening sites. Molds are important in the development of flavor, but some fungi of the genera *Aspergillus* and *Penicillium* can produce undesirable mycotoxins [65]. For example, a combination of high moisture, low temperature and the predominance of penicillia has been found to be conducive to the production of penicillic acid, a carcinogen [66]. The filamentous fungus found at the highest abundance was *Aspergillus cibarius* in L02, L11 and L12 (3.38%, 42.51%, and 35.30%, respectively) followed by *Penicillium thymicola* (r.a. 8.78% in L12), and *Geotrichum candidum* (r.a. 4.90% in L04). *Aspergillus* and *Penicillium* are the main genera found in fermented sausages, while *Geotrichum* is found with lower frequency [6,51,67,68,69].

## 4. Conclusions

The present study suggests that LAB may provide a larger contribution to prevent pathogen growth than ripening time and/or physicochemical characteristics (pH, moisture, NaCl, and a_w_) in dry-fermented, low-acid artisanal Colonial sausages. Although we provide some evidence for a protective role of LAB, and enteric zoonotic viruses (HEV and RV-A) were not detected in the sausage samples, the presence of *Cl. perfringens* is an indication of the need for sanitary improvements in the production process to prevent foodborne outbreaks.

## Figures and Tables

**Table 1:** Origin of Colonial salami-type dry-fermented sausages in Santa Catarina State, ripening time and physical-chemical characteristics.

Sample	City	Ripening (Days)	Casing	pH	Moisture (%)	NaCl (%)	WPS (%)	a_w_
L01	Piratuba	40	N	5.50	30.02	4.30	12.50	0.891
L02	Herval Velho	16	S	6.30	50.53	3.70	6.80	0.950
L03	Jaborá	1	S	6.10	51.14	3.17	5.80	0.957
L04	Concórdia	7	S	5.10	30.20	6.15	16.90	0.829
L05	Luzerna	2	N	5.80	50.22	4.20	7.70	0.943
L06	Videira	9	N	5.50	41.03	4.32	9.50	0.927
L07	Salto Veloso	12	N	6.40	40.85	4.75	10.40	0.917
L08	Salto Veloso	6	N	5.60	38.10	3.01	7.30	0.946
L09	Tangará	6	N	6.00	56.69	4.18	6.90	0.949
L10	Caçador	16	N	6.00	28.48	5.04	15.00	0.857
L11	Videira	2	N	6.00	43.22	4.49	9.40	0.928
L12	Iomerê	10	N	5.70	39.02	4.92	11.20	0.908
L13	Lacerdópolis	7	S	5.40	39.06	4.41	10.10	0.920

WPS: water phase salt; a_w_: water activity; N: natural; S: synthetic.

**Table 2:** Microbial analyses of Colonial salami-type dry-fermented sausages.

Sample	TC *	TTC *	CPS *	SRC *	LAB *	Enterococci *	Yeasts *	Molds *	*Salmonella* **	*Listeria* spp. **
L01	2.04	2.00	2.00	1.00	7.60	5.89	8.34	<2.00	A	P
L02	<1.00	<1.00	<2.00	<1.00	6.91	4.29	6.64	7.00	A	P
L03	1.70	1.70	<2.00	<1.00	8.32	3.11	3.11	7.30	A	A
L04	3.89	3.81	<2.00	1.48	6.60	5.68	6.81	4.30	A	A
L05	4.73	4.73	2.48	<1.00	7.84	5.78	8.30	7.48	A	P
L06	2.26	<1.00	<2.00	<1.00	7.84	3.63	7.28	7.00	A	A
L07	1.00	1.00	4.73	<1.00	7.86	6.20	6.81	7.30	A	A
L08	<1.00	<1.00	<2.00	<1.00	7.91	3.67	6.78	7.00	A	A
L09	2.08	1.60	2.90	<1.00	8.38	6.25	7.20	4.48	A	A
L10	<1.00	<1.00	<2.00	<1.00	6.79	3.15	6.55	7.30	A	A
L11	2.86	2.28	2.00	<1.00	6.75	4.70	7.47	7.30	A	A
L12	2.00	2.00	2.30	<1.00	8.48	3.87	7.95	4.00	A	P
L13	<1.00	<1.00	<2.00	1.00	7.96	2.30	6.83	<2.00	A	P

* cfu·log^−1^; ** Presence (P) or absence (A) in 25 g of sausage; TC: total coliforms; TTC: thermotolerant coliforms; CPS: coagulase-positive staphylococci; SRC: sulfite-reducing clostridia; LAB: lactic acid bacteria.

## Data Availability

The Data associated with this article can be requested by e-mail.
